# Baltimore College of Dental Surgery

**Published:** 1881-03

**Authors:** 


The Baltimore College of Dental Surgery held its Forty-first Annual Commencement on the 2nd instant, and granted its Diploma to fifty-three graduates. The address to the graduating class was delivered by Rev. Dr. Kirkus, and was replete with good advice to the young Dentists.

